# Stimulation of the Semicircular Canals or the Utricles by Clinical Tests Can Modify the Intensity of Phantom Limb Pain

**DOI:** 10.3389/fneur.2019.00117

**Published:** 2019-02-26

**Authors:** Catalina Aranda-Moreno, Kathrine Jáuregui-Renaud, Jaime Reyes-Espinosa, Angelina Andrade-Galicia, Ana E. Bastida-Segura, Lourdes G. González Carrazco

**Affiliations:** ^1^Unidad de Investigación Médica en Otoneurología, Instituto Mexicano del Seguro Social, Mexico City, Mexico; ^2^Hospital General de Zona 1“A”, Instituto Mexicano del Seguro Social, Mexico City, Mexico; ^3^Unidad de Medicina Familiar 70, Instituto Mexicano del Seguro Social, Mexico City, Mexico; ^4^Hospital General de Zona 47, Instituto Mexicano del Seguro Social, Mexico City, Mexico; ^5^Hospital General de Zona 8, Instituto Mexicano del Seguro Social, Mexico City, Mexico

**Keywords:** vestibular, otoliths, phantom limb pain, despersonalization, body image

## Abstract

**Background:** After amputation, phantom limb pain may be produced by the multisensory processes underling the experience of an intact body. Clinical evidence has shown that cold caloric vestibular stimulation may modify the perception of phantom limb pain. However, it is yet unknown if this effect can be observed after the mild vestibular stimulation given by the clinical caloric test, or after utricle stimulation by centrifugation. Additionally, there are no studies on the association between the report of altered perceptions or experience of the self or the environment (depersonalization/derealization symptoms) and phantom limb pain.

**Objective:** To assess the influence of unilateral stimulation of the horizontal semicircular canals by clinical caloric test, and the utricles by unilateral centrifugation on the intensity of phantom limb pain, and to explore the association between phantom limb pain and symptoms of depersonalization/ derealization.

**Methods:** 34 patients (56 ±7 years old, 23 men) accepted to participate after 3 to 23 months of unilateral supracondylar amputation, secondary to type 2 diabetes mellitus. After assessment of vestibular function and symptoms of common mental disorders, using a cross-over design, in 2 separate sessions with 1 week in between, vestibular stimulation was delivered by right/left caloric test (30 or 44°C) or right/ left centrifugation (3.85 cm, 300°/s peak). Before and after each vestibular stimulus, the intensity of phantom limb pain and depersonalization/derealization symptoms were assessed, with a daily follow-up of pain intensity during 1 week.

**Results:** Either caloric stimulation or unilateral centrifugation decreased phantom limb pain (*p* < 0.05), along with decrease of symptoms of depersonalization/derealization (*p* < 0.05). One third of the patients reporting pain decrease immediately after stimulation also reported no pain at least for 1 day.

**Limitations:** No sham condition was included.

**Conclusions:** Vestibular stimulation by the clinical caloric tests or by unilateral centrifugation may decrease the intensity of phantom limb pain, with decrease of perceptions of unreality. These effects might be related to an update of the immediate experience of the body, given by the sensory mismatch induced by asymmetrical vestibular stimulation.

## Introduction

Perception of head acceleration and orientation in space is sustained by right/left asymmetry of the input from each of the bilateral 5 vestibular organs, with crossing inhibitory/excitatory connections in the vestibular pathway; the three semicircular canals measure how the head rotates in space, while the utricle and the saccule measure how the body translates in space, and how it is positioned relative to gravity (1, 2).

Until the twenty-first century, selective stimulation of each vestibular organ was feasible just at some research facilities. Currently, the most widely used method to stimulate and assess the vestibular function is to modify the spontaneous discharge from each (right or left) horizontal semicircular canal by the clinical caloric tests, where water either at 44°C (excitatory) or at 30°C (inhibitory) is introduced into the external ear canal, with the head positioned 30°arc from the earth horizontal (3). Also, to modify the spontaneous discharges of both ears simultaneously, rotational testing is performed by active or passive movements of the head only or the whole body (4). In addition, mainly for research, galvanic vestibular stimulation is used to stimulate the entire vestibular nerve, via polarization effects (5).

However, since human beings have evolved under the gravitational field, gravity is central to orient the body as well as the objects in space (6). In the last two decades, significant advancements have been made for the assessment of the graviceptors (4). Among other tests of the utricular function, unilateral centrifugation on a rotating chair can be performed when the subject is shifted either to the right or to the left. Then, the center of rotation is located directly over one utricle, while a centripetally displaced g-force is applied over the eccentrically displaced utricle, with a sheering effect as if the displaced utricle was undergoing a static head tilt (7).

Pain is an interpretation of nociceptive input, influenced by memories, emotional, pathological, genetic, and cognitive factors (8). Phantom limb pain refers to the pain perceived in a part of the body that is no longer present. Independently from the general characteristics of the patients, a combination of cortical and peripheral mechanisms may interact to result in the experience of phantom limb pain (9). Evidence suggest that phantom limb pain emerges through altered afferent input from the affected limb and dorsal root ganglia, together with disrupted sensory processing and derangement of body representation at the supra-spinal and cortical level [for review see (10, 11)].

Body image and body schema are terms used to describe the body representation. The body image refers to the concept of the shape, the size and the mass of the body and its parts (12); while the body schema can be defined as a dynamic representation of the relative positions of the body parts derived from multiple sensory and motor inputs (e.g., proprioceptive, vestibular, tactile, visual, efference copy) that interacts with motor systems for movement and action (13, 14). Behavioral studies demonstrate that vestibular signals, including the graviceptors, contribute to continuously update the body schema and to control the interactions with objects in the environment [for review see (15)]. Consistently, vestibular stimulation in healthy subjects and vestibular disease in patients may trigger feelings of unreality of both the body and the environment (depersonalization/derealization symptoms) (16–18).

In healthy subjects, experimental evidence has shown that semicircular canal stimulation may change the instantaneous representation of the body segments (19). Also, in microgravity, mental transformation of one's own body or body parts becomes more difficult (20). In patients, caloric vestibular stimulation, rotation or galvanic vestibular stimulation may modify certain illusions of body representation, such as somatophrenia (a tendency to imagine or exaggerate body ills), hemi-body neglect, or phantom limb (21–23). In amputees, caloric vestibular stimulation may even evoke phantom limb illusion (24).

Caloric vestibular stimulation may also reduce experimental pain (25, 26) as well as clinical central pain (27, 28). In healthy subjects cold left caloric vestibular stimulations may elicit a modulation of both nociceptive processing and pain perception. Using laser pulses for selective stimulation of the left hand skin nociceptors, before and after left cold caloric vestibular stimulation, showed that vestibular stimulation induced a transient decrease of subjective pain intensity, which was associated with reduced amplitude of all laser evoked potential components, including the first arrival of nociceptive input to primary somatosensory cortex (26). In several chronic pain conditions, caloric vestibular stimulation may temporarily ameliorate pain (29). It may decrease chronic central post-stroke pain, along with reduction of somatic delusions (30). In 2 of 4 patients with pain following spinal cord injury, caloric vestibular stimulation had an analgesic effect (31). In a group of 10 patients with phantom limb pain, caloric vestibular stimulation was related to pain reduction in all of them (24). In patients with a variety of pain conditions including phantom limb pain, spinal cord injury and complex regional pain, a significant analgesic effect was observed after cold caloric vestibular stimulation, compared to a control stimulation (ice-pack to forehead) (32).

The effect of vestibular stimulation on nociception has been usually assessed by strong cold stimulation of the left horizontal semicircular canal. However, irrigation of the external ear canal with a strong cold stimulus can be painful, and it could activate inhibitory nociceptive pathways (33). Yet, in patients with limb amputation, a moderate cold stimulus with water at 20°C have been used to evoke phantom perception as well as to decrease phantom limb pain, either ipsilateral or contralateral to the amputation side, supporting that caloric stimulation seems to have general activation effects on the neural mechanisms underpinning the representation of the body (24). This finding suggests that the mild caloric vestibular stimulation used in the clinical setting could have an effect on phantom limb pain. In addition, the influence of altered graviception by utricle stimulation on the perception of phantom limb pain has not been assessed. Moreover, there is a lack of information on the possible association between the perception of phantom limb pain and altered perceptions or experience of the self or the environment (symptoms of depersonalization/derealization).

The Aims of the Present Study Were:

- To assess if the mild caloric stimulation of the horizontal semicircular-canals, given by any of the stimuli comprising the clinical caloric tests, could have an effect on the intensity of phantom limb pain similar to the effect already reported for cold caloric vestibular stimulation.
- To assess if utricular stimulation by unilateral centrifugation could have an effect on the intensity of phantom limb pain, similar to the effect of caloric stimulation.
- To explore the association between changes on phantom limb pain and the report of altered perceptions or experience of the self or the environment, by probing for depersonalization/derealization symptoms simultaneously with phantom pain intensity, just before and after vestibular stimulation.

In order to partially control for inter-subject variability, the study was performed in a homogeneous group of patients, all of them had unilateral supracondylar amputation, secondary to complications of type 2 diabetes mellitus, and a cross over design was used to test semicircular canal stimulation and utricle stimulation, with a week in-between, including a daily follow-up of pain intensity after each vestibular stimulus.

## Materials and Methods

### Participants

The research protocol was approved by the Research and Ethics Committees of the Institution (IMSS. R-2015-785-050).The study was carried out in accordance with the Declaration of Helsinki and its amendments.

Thirty four patients (56 ± 7 years old, mean ± standard deviation; 23 men) gave their informed consent to participate in the study. All of them reported phantom limb pain after 3–23 months of unilateral supracondylar amputation (median 5 months) (Table 1). None of them wore prosthetic devices, or had history of otology or balance disorders or prolypherative retinopathy or advanced renal disease. Patients with a history of migraine or other neurological or psychiatric disorders (submission to psychiatric care or psychopharmacological treatment) were not included in the study. Three patients received the first vestibular stimuli, but did not come back for the second stimuli due to unavailability of adequate transportation or personal circumstances unrelated to the study or the phantom limb pain.

**Table 1:** General characteristics of 34 patients with unilateral supracondylar amputation of a lower limb, secondary to type 2 diabetes mellitus.

**CHARACTERISTIC**	
Amputated limb (right/left)	18/16
Handiness (right/left)	32/2
**LATTINEN SCORE (RANGE & MEDIAN)**	
Total score	2–13 (7)
Pain intensity	1–3 (1.5)
Pain frequency	1–4 (2)
Need of medication	0–3 (1)
Handicap	0–3 (1)
**FEATURES OF PHANTOM PAIN (FREQUENCY)**	
Electric shocks	91%
Painful cold	50%
Burning	20%
**ASSOCIATED SYMPTOMS (FREQUENCY)**	
Pins & needles	76%
Numbness	76%
Tingling	73%
Itching	50%
**SYMPTOMS OF COMMON MENTAL DISORDERS(FREQUENCY)**	
GHQ12 score ≥3	47%
Zung anxiety score ≥45	17%
Hamilton score ≥8	82%
Dissociative experiences score ≥8	61%

The sample size was calculated in order to identify the already reported general effect of caloric vestibular stimulation on phantom limb pain, with a pain intensity decrease in 90% of the participants, precision of ±0.10 and 2 sided type I error of 0.01.

### Procedures

#### Evaluations Prior to Vestibular Stimulation

The diagnosis of phantom limb pain was confirmed by an independent surgeon within the week before vestibular stimulation. Then, adequate ear function was verified by quantitative testing, using tympanometry (Interacoustics AT235, Assens), audiometry (Orbiter 922 Madsen, Otometrics, Taastrup), eye movement recordings and rotational tests at 0.16 Hz and 1.28 Hz (I-Portal NOTC, Neuro Kinetics, Pennsylvania). Within the same day of the first vestibular stimulation, pain characteristics were assessed using the Lattinen index (34) and the DN4 questionnaire (*Douleur Neuropathique 4 Questions*) (35); and symptoms of common mental disorders were evaluated using the General Health Questionnaire of 12 items (36), the Zung Instrument for Anxiety Disorders (37), the 17-items Hamilton Depression Rating Scale (38), and the Dissociative Experiences Scale (39). Additionally, handiness was assessed by the Edinburg inventory (40).

The Lattinen Index is a tool for measuring chronic pain. It comprises 4 dimensions: Pain intensity, Pain frequency, Analgesic consumption, Functional Ability, and Hours of Sleep. In Spanish, the overall score as well as the individual dimensions have been validated, showing positive correlation with the Visual Analog Scale and the McGill Pain Questionnaire, among other scales. The internal consistency and test-retest assays have shown coefficient values of alpha > 0.7 and intra-class correlation > 0.85, respectively (34).

The DN4 is a questionnaire for identification of chronic pain associated to a lesion in the nervous system. It includes 10 items. The first seven items are related to the quality of pain (burning, painful cold, and electric shocks) and its association to abnormal sensations (tingling, pins and needles, numbness, and itching). The other 3 items are related to neurological examination in the painful area (touch hypoesthesia, pinprick hypoesthesia and tactile allodynia) (35).

The 12 item General Health Questionnaire (GHQ-12) comprises 12 items to identify symptoms of depression and anxiety. It was scored using the “GHQ method” of 0-0-1-1 (range 0–12) (36).

The Zung Instrument for Anxiety Disorders is a 20-item scale, with some of the items keyed positively and some negatively, on a four-point scale ranging from 1 “none or a little of the time” to 4 “most or all of the time.” The final score range from 20 to 80, a score between 20 and 44 is considered in the normality range (37).

The 17 item-Hamilton Depression Rating Scale evaluate depressed mood, vegetative and cognitive symptoms of depression, and co-morbid anxiety symptoms (23, 24). The 17 items were rated on a 5-point (0–4) with a rating of 0 = absent; 1 = doubtful to mild; 2 = mild to moderate; 3 = moderate to severe; 4 = very severe. The final score range from 0 to 48, a score between 0 and 7 points is considered in the normality range (38).

The Dissociative Experiences Scale comprises a broad range of dissociative experiences including disturbances in memory, identity, and cognition, and feelings of derealization, depersonalization, absorption, and imaginative involvement. Scores on each of the 28 items could range from 0%, “This never happens to you,” to 100%, “This always happens to you,” using multiples of ten (e.g. 10, 20, 30%,…). The total score is calculated by dividing the sum of the individual scores by 28 (range 0 to 100%). A cutoff of 8 is considered in the low normal range (39).

#### Vestibular Stimulation

A cross over design was used to administer 2 vestibular stimuli by an independent investigator, with a follow-up of pain intensity for 7 days after each stimulus. Since the effect of vestibular stimulation was expected to be transitory, the follow-up was intended just to verify the return to baseline before the next stimulation.

During the first visit, patients were assigned by a random number list either to caloric stimulation of the right or the left horizontal semicircular canal, at 30°C or 44°C (20) (ICS NCI 480, Otometrics, Taastrup), or to unilateral centrifugation at 3.85 cm right or left (300°/s peak velocity; I-Portal NOTC, Neuro Kinetics, Pennsylvania). During the second visit, patients who already received caloric stimulation were assigned to centrifugation and *visceversa*, with random stimulation of the right or the left ear. The intensity of phantom limb pain, by the pain intensity subs-core of the Lattinen Index (34), and depersonalization/derealization symptoms (41) were assessed before and after delivering vestibular stimulus.

After caloric vestibular stimulation all participants showed horizontal nystagmus and reported vertigo; while during centrifugation, the deviation of the visual vertical was consistent with the side of the stimulus.

After each stimulation session, patients received instructions to daily record the intensity of phantom pain on a printed version of the pain intensity sub-score of the Lattinen Index for each day, and every day, they received a standardized phone call remaining them to register the intensity of pain.

To facilitate self-report of pain intensity, since the selected type of patients usually has a variety of visual deficiencies, the pain intensity dimension of the Lattinen Index was preferred among other instruments. This subs-core includes both numeric and simple descriptors that are organized vertically, and it is rated on a 5-point scale from 0 to 4, with a rating of 0 = no; 1 = mild; 2 = moderate; 3 = severe; 4 = unbearable (34).

The 28 item depersonalization/derealization inventory is a tool designed to assess symptoms of depersonalization/derealization in clinically anxiety states, more than in a dissociative disorders context. The severity of each item is coded on a scale where 0 = does not occur, 1 = mild, 2 = moderate, 3 = severe and 4 = very severe. The total score is calculated by adding-up all the points (range 0–112). The higher scores are related to a higher frequency and/or severity of the symptoms, no cutoff score has been suggested (41).

### Analysis

Statistical analysis was performed on coded data to assess the immediate responses to vestibular stimulation and the effect of confounding variables, using paired “t” test, Cohen's h and Cohen's d, and analysis of covariance (CSS, Statsoft, Tulsa), with a 2 sided significance level of 0.05. In addition, since just before vestibular stimulation, some patients reported the absence of phantom pain, discriminant function analysis (CSS, Statsoft, Tulsa) was used to assess the association between the report of specific symptoms of depersonalization/ derealization and the report of phantom limb pain.

Discriminant function analysis is used to determine which variables discriminate between two or more naturally occurring groups. In this study, it was used to determine which symptoms of depersonalization/derealization could discriminate between patients with/without phantom limb pain just at the moment of vestibular stimulation, as well as between those who reported or not a decrease of phantom limb pain after stimulation.

## Results

### Phantom Limb Pain

The characteristics of phantom limb pain reported by the patients are described in Table 1. Although, all the patients reported phantom limb pain during the clinical evaluation, at the moment of the first vestibular stimulation, 28 patients reported pain and 6 had no pain. At the moment of the second vestibular stimulation (day 8), 11 patients reported phantom limb pain, 20 patients had no pain and 3 patients did not come back.

According to the type of stimuli, since 3 patients received just one stimulus, 32 patients received caloric stimulation (8 right/8 left at 30°C; 7 right/9 left at 44°C) (Table 2), and 33 patients received centrifugation (19 right/14 left) (Table 2). After any stimuli, there was a decrease of pain intensity, with a very large size effect (Table 2). However, among those who received right caloric stimulation at 44°C, just one patient reported phantom limb pain at the moment of vestibular stimulation, which decreased after the stimulus, while the other 6 patients reported no pain at the moment of vestibular stimulation.

**Table 2:** Mean and standard deviation of the mean of the sub-score of pain intensity (from 0 to 4) of the Lattinen index, reported by the patients with pain before and after each type of vestibular stimulation.

**Vestibular stimuli**	**Number**	**Pain intensity score**		***p***	**Effect size**
		**Before**	**After**		**Cohen's d**
**CALORICS**					
Right 30°C	7	1.2 ± 0.4	0.5 ±0.7	**0.008**	1.75
Left 30°C	6	1.5 ±0.8	0	**0.007**	1.87
Right 44°C	1	1	0	NA	NA
Left 44°C	5	1.6 ± 0.5	0.2 ±0.4	**0.025**	2.8
**CENTRIFUGATION**					
Right	11	1.4 ±0. 6	0.2 ±0.4	**0.001**	2
Left	9	1.7 ±0.8	0.5 ±1	**0.002**	1.5

*Significant values are given for “t” test for dependent samples. The p values > 0.05 are highlighted*.

**Figure 1:**
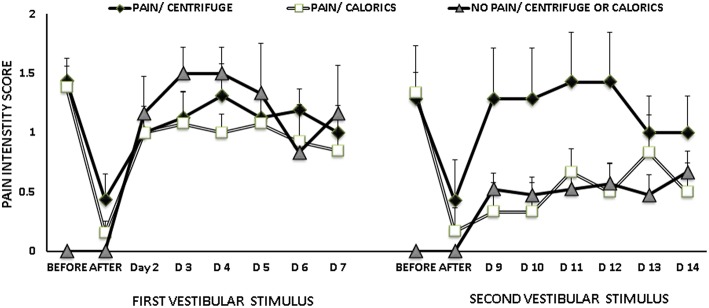
Mean and standard error of the mean of pain intensity scores, before and after vestibular stimulation, according to the report of phantom limb pain at the moment of vestibular stimulation and the type of stimuli, either caloric stimulation or unilateral centrifugation. At the second stimulation, patients who already received caloric stimulation were assigned to unilateral centrifugation and *visceversa*. At the first vestibular stimulation, among 28 patients with phantom pain 13 received caloric stimulation and 15 received unilateral centrifugation, while 6 patients reported no phantom pain. At the second vestibular stimulation, among 11 patients with phantom pain 5 received caloric stimulation and 6 received unilateral centrifugation, while 20 patients reported no phantom pain.

The frequency of pain decrease after either caloric stimulation or centrifugation was similar (Table 2), the size effect between the 2 stimuli was small (Cohen's h = 0.35). The first time, 92% (12/13) and 80% (12/15) of the patients reported pain decrease after caloric stimulation and centrifugation, respectively, the 6 patients with no pain reported no change (Figure 1). The second time, 80% (4/5) and 66% (4/6) of the patients reported pain decrease after caloric stimulation and centrifugation respectively, the 20 patients with no pain reported no change (Figure 1). The two times, one third of the patients who reported pain decrease immediately after stimulation had no pain at least for 1 day (Figure 1).

**Figure 2:**
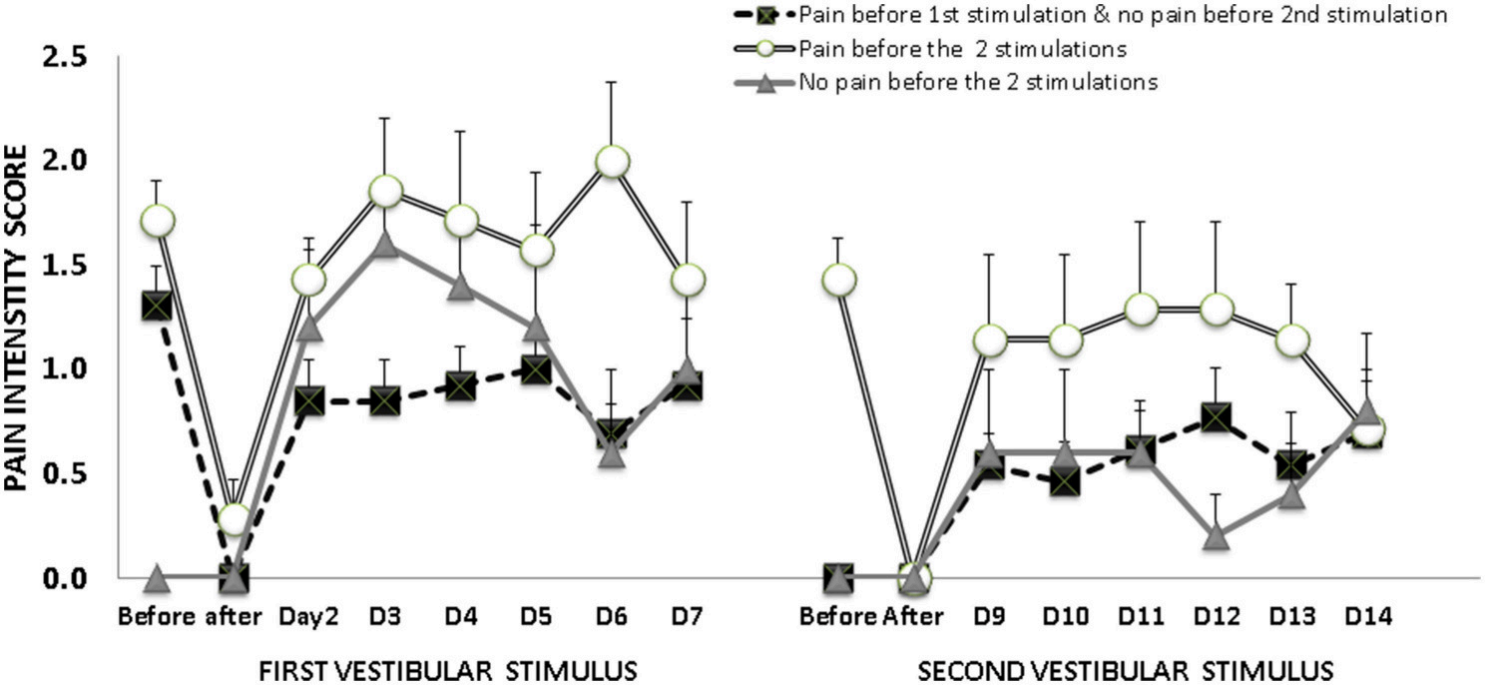
Mean and standard error of the mean of pain intensity scores, before and after vestibular stimulation, according to the report of phantom pain at the moment of each vestibular stimulation 13 patients reported phantom pain just before the first vestibular stimulation, but no phantom pain before the second stimulation; while 7 patients reported phantom pain before the 2 stimulations and 5 patients reported no phantom pain before either stimulation.

According to the report of phantom limb pain before and after vestibular stimulation, pain intensity scores are shown in Figure 2. Among the 24 patients who reported pain decrease after the first vestibular stimulation, 21 patients received the second vestibular stimulation, of whom 13 patients reported no pain and 8 reported pain. The 3 patients who received just the first vestibular stimulation reported pain decrease after stimulation, which lasted for 1 or 2 days. At the moment of the 2 vestibular stimulations, 5 patients reported no phantom limb pain. Their general characteristics were similar to the characteristics of the whole group of patients. Contrary, two patients reported persistent phantom limb pain, before and after the 2 vestibular stimulations. They were women aged 53 and 58 years, with recent amputation (3 and 4 months, 1 right/1 left); the 2 of them had a GHQ12 score ≥3, with symptoms suggestive of depression, and a dissociative experiences score ≥8.

The frequency of symptoms of common mental disorders is described in Table 1. Multivariate analysis showed no influence of the report of symptoms of common mental disorders on the Lattinen Index total score attained at the clinical evaluation, or the pain intensity sub-score reported before any of the 2 vestibular stimuli (*p* > 0.05). However, these results may have been influenced by the low frequency of symptoms of anxiety, and the high frequency of symptoms of depression that were reported by the participants.

### Symptoms of Depersonalization/Derealization

Depersonalization/derealization symptoms reported by the patients are described in Table 3.

**Table 3:** Frequency and score range of each of the depersonalization/derealization symptoms (42) reported by the patients before and after vestibular stimulation.

**Depersonalization/** **derealization symptoms**	**Centrifuge (*****N*** **=** **33)**		***p***	**Calorics (*****N*** **=** **32)**		***p***
	**Before frequency (score range)**	**After frequency (score range)**		**Before frequency (score range)**	**After frequency (score range)**	
1. Surroundings seem strange and unreal	33.3% (0–3)	30.3% (0–3)	0.17	37.5% (0–3)	31.3% (0–3)	0.5
2. Time seems to pass very slowly	54.5% (0–4)	45.5% (0–2)	**0.02**	53.1% (0–3)	43.8% (0–3)	0.14
3. Body feels strange or different in some way	78.8% (0–4)	57.6% (02)	**0.0007**	81.3% (0–4)	53.1% (0–3)	**0.005**
4. Feel like you've been here before (déjà vu)	18.2% (0–2)	15.2% (0–2)	0.74	21.9% (0–3)	25.0% (0–3)	0.82
5. Feel as though in a dream	30.3% (0–4)	51.5% (0–2)	0.72	40.6% (0–4)	40.6% (0–3)	0.30
6. Body feels numb	69.7% (0–4)	48.5% (0–2)	**0.0004**	68.8% (0–3)	56.3% (0–3)	0.19
7. Feeling of detachment or separation from surroundings	33.3% (0–3)	21.2% (0–2)	**0.02**	43.8% (0–4)	15.6% (0–2)	**0.009**
8. Numbing of emotions	54.5% (0–3)	48.5% (0–2)	0.13	59.4% (0–3)	37.5% (0–2)	**0.007**
9. People and objects seem far away	30.3% (0–3)	18.2% (0–2)	**0.03**	40.6% (0–4)	25.0% (0–2)	0.07
10. Feeling detached or separated from your body	27.3% (0–4)	12.1% (0–3)	**0.02**	40.6% (0–4)	25.0% (0–2)	**0.02**
11. Thoughts seem blurred	30.3% (0–2)	36.4% (0–2)	0.78	50.0% (0–3)	28.1% (0–2)	**0.005**
12. Events seem to happen in slow motion	15.2% (0–2)	48.5% (0–2)	**0.005**	31.3% (0–3)	21.9% (0–3)	0.16
13. Your emotions seem disconnected from yourself	36.4% (0–4)	18.2% (0–2)	**0.03**	43.8% (0–3)	28.1% (0–3)	**0.02**
14. Feeling of not being in control of self	39.4% (0–2)	27.3% (0–2)	0.32	46.9% (0–3)	25.0% (0–2)	**0.02**
15. People appear strange or unreal	15.2% (0–1)	6.1% (0–1)	0.08	21.9% (0–2)	6.3%% (0–2)	0.09
16. Dizziness	39.4% (0–2)	60.6% (0–4)	0.06	40.6% (0–2)	71.9% (0–3)	0.09
17. Surroundings appear covered with a haze	18.2% (0–2)	33.3% (0–1)	0.21	28.1% (0–3)	25.0% (0–2)	0.35
18. Vision is dulled	75.8% (0–4)	63.6% (0–3)	**0.01**	81.3% (0–4)	68.8% (0–3)	0.11
19. Feel as if walking on shifting ground	21.2% (0–2)	24.2% (0–2)	0.74	21.9% (0–2)	18.8% (0–3)	0.78
20. Difficulty understanding what others say to you	48.5% (0–4)	18.2% (0–1)	**0.0004**	50.0% (0–3)	37.5% (0–2)	0.16
21. Difficulty focusing attention	33.3% (0–3)	21.2% (0–2)	0.09	46.9% (0–3)	34.4% (0–2)	**0.02**
22. Feel as though in a trance	27.3% (0–2)	36.4% (0–2)	0.18	18.8% (0–3)	21.9% (0–1)	0.71
23. The distinction between close and distant is blurred	60.6% (0–4)	45.5% (0–2)	**0.008**	59.4% (0–3)	50.0% (0–2)	**0.04**
24. Difficulty concentrating	45.5% (0–3)	24.2% (0–1)	**0.016**	46.9% (0–3)	37.5% (0–2)	0.13
25. Feel as though your personality is different	51.5% (0–3)	42.4% (0–3)	0.21	56.3% (0–3)	34.4% (0–2)	**0.001**
26. Feel confused or bewildered	36.4% (0–3)	30.3% (0–3)	0.49	43.8% (0–3)	28.1% (0–2)	**0.02**
27. Feel isolated from the world	36.4% (0–3)	15.2% (0–1)	**0.007**	43.8% (0–4)	28.1% (0–1)	**0.01**
28. Feel “spacy” or “spaced out”	9.1% (0–1)	12.1% (0–3)	0.25	15.6% (0–3)	15.6% (0–1)	0.42

*Significant values are given for “t” test for dependent samples. The p values > 0.05 are highlighted*.

After the first vestibular stimulation, the total score of depersonalization/derealization symptoms decreased in all patients (18 ± 15 vs. 10 ± 8; Cohen's d = 0.53) (paired “t” test, *t* = 3.4, *p* = 0.001), including those who had pain (18 ± 14 vs. 10 ± 6; Cohen's d = 0.57) (paired “t” test, *t* = 3.2, *p* = 0.003). After the second vestibular stimulation the score also decreased in all patients (16 ± 13 vs. 11 ± 10; Cohen's d = 0.38) (paired “t” test, *t* = 3.0, *p* = 0.005), but almost significantly in those who had pain (19 ± 13 vs. 12 ± 8; Cohen's d = 0.53) (paired “t” test, *t* = 2.15, *p* = 0.056).

Before any stimulation, the symptom “Body feels strange or different in some way” was reported by circa 80% of all the patients, which decreased after either stimulus to circa 55% (“t” test, after either stimulus *t* ≥ 2.9, *p* ≤ 0.005). Other symptoms that decreased after either stimulus were: “Feeling of detachment or separation from surroundings,” “Feeling detached or separated from your body,” “Your emotions seem disconnected from yourself,” “The distinction between close and distant is blurred,” and “Feel isolated from the world” (“t” test, after either stimulus *t* ≥ 2.3, *p* ≤ 0.02).

Discriminant function analysis showed that the combination of the symptoms “Body fells numb,” “Vision is dulled,” and “Feel as though in a trance,” before vestibular stimulation, discerned 81% of the times those who had phantom pain, and 50% of those with no pain (Malahanobis distance 1.07, *F* = 5.2, *p* = 0.002). In addition, “Feel as though in a trance,” before vestibular stimulation, discerned 82% of the times the decrease of the intensity of phantom pain after vestibular stimulation and 57% of no decrease (Malahanobis distance 1.13, = 5.6, *p* = 0.02).

## Discussion

In this study, mild caloric stimulus of the horizontal semicircular canals, by clinical test, as well as unilateral stimulation of the utricles, by centrifugation, were related to temporary decrease of phantom limb pain, in association to decrease on the report of symptoms of depersonalization/ derealization.

The finding that stimulation of either the semicircular canals or the utricles (right or left) may have an effect on phantom limb pain, further supports the hypothesis by Andre et al. (24), that vestibular stimulation may have general effects on the neural mechanisms underpinning the representation of the body. However, to assess this hypothesis, further functional imaging studies on multisensory integration are needed, taking into account the effect of magnetic vestibular stimulation (42). In addition, it is important to ponder that, in this study, the stimulation provided by the two stimuli was asymmetric and not physiological. During unilateral caloric stimulation each labyrinth is activated separately; while during eccentric acceleration in a fixed earth vertical attitude, the fast rotation of the direction of the resultant linear acceleration is not accompanied by a tilt velocity signal of the semicircular canals [for review see (43)]. Then, the sudden discrepancy between the discordant sensory input and the reference frame given by individual experiences could also have had an influence on the change of the immediate experience of the body in the environment.

Interestingly, the report of general feelings of unreality, like “Feel as though in a trance” showed the greatest association with the report of phantom limb pain before stimulation, as well as with pain decrease after vestibular stimulation. The biological viability of these associations may be supported by the contribution of the vestibular inputs to the conscious experience of the body (19). Since the vestibular system is phylogenetically ancient, and its connectivity has prevailed in many networks, contributing to the internal representation of the self (44, 45); while, nociception contributes to subtend the most primitive forms of somatosensation (46), and it also contributes to the multisensory representations that underlie the sense of one's own body and of peripersonal space (25).

In this study, participants were asked to report phantom limb pain intensity immediately before and after vestibular stimulation, with a daily follow-up. Patients with no pain just at the moment of vestibular stimulation reported no change after stimulation, but during the following days they reported their usual experience of phantom pain. Then, half of the patients experienced phantom limb pain only before one vestibular stimulation, which decreased after stimulation, and they reported no pain and no change after the other vestibular stimulation. This finding advocates for an authentic report of phantom limb pain before and after vestibular stimulation. Of note, just 16% of the patients reported no pain before the two vestibular stimulations. This fluctuation of pain intensity is consistent with epidemiological studies showing that patients with persistent phantom limb pain may report that pain is usually intermittent (47, 48). In a survey of 255 lower extremity amputees several months or years after amputation, 81% of those reporting phantom limb pain stated that it was episodic in nature (49). Similarly, in a group of 92 patients with lower extremity amputation only 37% of the group who reported phantom limb pain experienced it more than half of the time (50).

Among the patients with phantom limb pain who reported pain decrease following the first vestibular stimulation, a week later, just 33% of them reported phantom limb pain again. This finding suggests that vestibular stimulation might also have an influence on the clinical evolution of phantom limb pain intensity. However, this study cannot test such hypothesis, which would require a different study design. On the other hand, 2 of the patients participating in the study reported persistent phantom limb pain, with no decrease after vestibular stimulation. Several factors may have conditioned the persistence of pain, including a possible influence of the distress related to grief (51). Also, in amputees, epidemiological evidence suggests that depressed mood may contribute to the experience of chronic pain, including phantom limb pain (52). Of note, all the participants of this study have type 2 diabetes mellitus, which doubles the odds ratio for comorbid depression (53).

The main limitation of the study is the lack of a sham stimulus. Since there was no previous study on the effect of mild stimulation of the semicircular canals by the stimuli comprising the clinical caloric tests or a possible effect of utricular stimulation on phantom limb pain, the study was designed to assess these effects in a selected group of patients with intricate diabetes complications. Then, care had to be taken to minimize exposure to conditions with uncertain benefit to the patient. Another limitation was the unsuitability of the majority of patients with type 2 diabetes mellitus to fulfill the stringent selection required to participate in the study, which limit the generalizability of the findings. Another limitation of the study was the failure to test the 4 stimuli included in the clinical caloric tests, since the majority of the patients exposed to warm caloric stimulus of the right ear reported no pain at the moment of stimulation. Then, the study cannot support or deny differences between warm stimulation of the right semicircular canal vs. any of the other 3 stimuli (right-cold, left-warm, & left-cold).

## Conclusion

The results show that the mild unilateral vestibular stimulation used for clinical tests, of either the horizontal semicircular canals or the utricles, might modify the intensity of phantom limb pain along with decrease on the report of altered perceptions or experience of the self or the environment. These effects might be related to an update of the immediate experience of the body, given by the sensory mismatch induced by asymmetrical vestibular stimulation.

## Data Availability

Full data is available on request to the corresponding author.

## Author Contributions

KJ-R: conceived and designed the study and the vestibular stimuli, analyzed and interpreted the data, and wrote the manuscript. CA-M: supervised the selection of participants, performed the stimuli, collected the data, and revised the manuscript. JR-E: selected and evaluated the participants and revised the manuscript. AA-G, AB-S, and LG: performed a preliminary assessment and invited the candidates to participate, and revised the manuscript.

### Conflict of Interest Statement

The authors declare that the research was conducted in the absence of any commercial or financial relationships that could be construed as a potential conflict of interest.

## References

[1] Fernandez C Goldberg JM. Physiology of peripheral neurons innervating otolith organs of the squirrel monkey. I. response to static tilts and to long-duration centrifugal force. J Neurophysiol. (1976) 39:970–84. 10.1152/jn.1976.39.5.970 824412

[2] Dieterich M Brandt T. The bilateral central vestibular system: its pathways, functions, and disorders. Ann NY Acad Sci. (2005) 1343:10–26. 10.1111/nyas.12585 25581203

[3] Hallpike CS. The Caloric Tests. J Laryngol Otol. (1956) 70:15–28. 10.1017/S0022215100052610 13278645

[4] Fife TD Tusa RJ Furman JM Zee DS Frohman E Baloh RW. Assessment: vestibular testing techniques in adults and children report of the therapeutics and technology assessment subcommittee of the American Academy of Neurology. Neurology (2000) 55:1431–41. 10.1212/WNL.55.10.1431 11094095

[5] Utz KS Dimova V Oppenländer K Kerkhoff G. Electrified minds: Transcranial direct current stimulation (tDCS) and Galvanic Vestibular Stimulation (GVS) as methods of non-invasive brain stimulation in neuropsychology—A review of current data and future implications. Neuropsychologia (2010) 48:2789–810 10.1016/j.neuropsychologia.2010.06.002 20542047

[6] Barra J Marquer A Joassin R Reymond C Metge L Chauvineau V Humans use internal models to construct and update a sense of verticality. Brain (2010) 133:3552–63. 10.1093/brain/awq311 21097492

[7] Clarke AH Schönfeld U Helling K. Unilateral examination of utricle and saccule function. J Vestib Res. (2003) 13:215–25. 15096665

[8] Tracey I Mantyh PW. The cerebral signature for pain perception and its modulation. Neuron (2007) 55:377–91. 10.1016/j.neuron.2007.07.012 17678852

[9] Nikolajsen J Jensen TS. Phantom limb pain. Brit J Anaesth. (2001) 87:107–16. 10.1093/bja/87.1.107 11460799

[10] Giummarraa MJ Moseley GL Phantom limb pain and bodily awareness: current concepts and future directions. Curr Opinion Anesth. (2011) 24:524–31. 10.1097/ACO.0b013e32834a105f 21772144

[11] Ramachandran VS Hirstein W. The perception of phantom limbs. Brain (1998) 121:1603–30. 10.1093/brain/121.9.1603 9762952

[12] Lukianowicz N. Body image disturbances in psychiatric disorders. Br J Psychiatry (1967) 113:31–47. 10.1192/bjp.113.494.31 6029368

[13] Head H Holmes G Sensory disturbances from cerebral lesions. Brain (1911) 34:102–254. 10.1093/brain/34.2-3.102

[14] Schwoebel J Boronat CB Branch Coslett H. The man who executed “imagined” movements: evidence for dissociable components of the body schema. Brain Cogn. (2002) 50:1–16. 12372347 10.1016/s0278-2626(02)00005-2

[15] Lenggenhager B Lopez C Vestibular Contributions to the Sense of Body, Self, and Others. In T. 16. 16. Cappon D, Banks R. Orientational perception, Arch Gen Psychiatry (1961) 5:88–100.

[16] Sang FY Jáuregui-Renaud K Green DA Bronstein AM Gresty MA. Depersonalisation/ derealisation symptoms in vestibular disease. J Neurol Neurosurg Psychiatry (2006) 77:760–6. 10.1136/jnnp.2005.075473 16464901 PMC2077438

[17] Aranda-Moreno C Jáuregui-Renaud K. Derealization during utricular stimulation. J Vestib Res. (2016) 26:425–31. 10.3233/VES-160597 28262646

[18] Metzinger Windt JM (Eds). Open MIND: 23(T). Frankfurt am Main: MIND Group (2015).

[19] Lopez C Schreyer HM Preuss N Mast FW. Vestibular stimulation modifies the body schema. Neuropsychologia (2012)50:1830–7. 10.1016/j.neuropsychologia.2012.04.008 22561888

[20] Grabherr L Karmali F Bach S Indermaur K Metzler S Mast FW. Mental own-body and body-part transformations in microgravity. J Vest Res. (2007) 17:279–87. 18626138

[21] Cappa S Sterzi R Vallar G Bisiach E. Remission of hemineglect and anosognosia during vestibular stimulation. Neuropsichologia (1987) 25:775–82. 10.1016/0028-3932(87)90115-1 3501552

[22] Karnath HO. Subjective body orientation in neglect and the interactive contribution of neck muscle proprioception and vestibular stimulation. Brain (1994) 117:1001–12. 10.1093/brain/117.5.1001 7953584

[23] Kerkhoff G. Modulation and rehabilitation of spatial neglect by sensory stimulation. Prog Brain Res. (2003) 142:257–71. 10.1016/S0079-6123(03)42018-9 12693266

[24] André JM Martinet N Paysant J Beis JM Le Chapelain L. Temporary phantom limbs evoked by vestibular caloric stimulation in amputees. Neuropsychiatry Neuropsychol Behav Neurol. (2001)14:190–6. 11513103

[25] Ferrè ER Bottini G Iannetti GD Haggard P. The balance of feelings: vestibular modulation of bodily sensations. Cortex (2013) 49:748–58. 10.1016/j.cortex.2012.01.012 22385524

[26] Ferrè ER Haggard P Bottini G Iannetti GD. Caloric vestibular stimulation modulates nociceptive evoked potentials. Exp Brain Res. (2015) 233:3393–401. 10.1007/s00221-015-4412-8 26282602 PMC4868137

[27] Ramachandran VS McGeoch PD Williams L Arcilla G. Rapid relief of thalamic pain syndrome induced by vestibular caloric stimulation. Neurocase (2007) 13:185–8. 10.1080/13554790701450446 17786778

[28] McGeoch PD Williams LE Lee RR Ramachandran VS. Behavioural evidence for vestibular stimulation as a treatment for central post-stroke pain. J Neurol Neurosurg Psychiatry (2008) 79:1298–301. 10.1136/jnnp.2008.146738 18550629

[29] Miller SM Ngo TT. Studies of caloric vestibular stimulation: implications for the cognitive neurosciences, the clinical neurosciences and neurophilosophy. Acta Neuropsychiatry (2007) 19:183–203. 10.1111/j.1601-5215.2007.00208.x 26952856

[30] Spitoni GF Pireddu G Galati G Sulpizio V Paolucci S Pizzamiglio L. Caloric vestibular stimulation reduces pain and somatoparaphrenia in a severe chronic central post-stroke pain patient: a case study. PLoS ONE (2016) 11:e0151213. 10.1371/journal.pone.0151213 27028404 PMC4814090

[31] Le Chapelain L Beis JM Paysant J André JM. Vestibular caloric stimulation evokes phantom limb illusions in patients with paraplegia. Spinal Cord (2001) 39:85–7. 10.1038/sj.sc.3101093 11402363

[32] Ngo T Barsdell WN Arnold CA Bedside neuromodulation of persistent pain and allodynia using caloric vestibular stimulation: an effectiveness trial. J Neurol Sci (2015) 357:e91 10.1016/j.jns.2015.08.304 PMC11505407 39457677

[33] LeBars D Villanueva L Bouhassira D Diffuse noxious inhibitory controls (DNIC) in animals and in man. Patol Fiziol Eksp Ter. (1992) 9:55–65. 1303506

[34] González-Escalada JR Camba A Muriel C Validación del índice de Lattinen para la evaluación del paciente con dolor crónico. Rev Soc Esp Dolor. (2012) 19:181–8. Available online at: http://scielo.isciii.es/pdf/dolor/v19n4/original2.pdf

[35] Bouhassira D Attal N Alchaar H Boureau F Brochet B Bruxelle J. Comparison of pain syndromes associated with nervous or somatic lesions and development of a new neuropathic pain dignostic questionnaire. Pain (2005) 114:29–36. 10.1016/j.pain.2004.12.010 15733628

[36] Goldberg DP Williams P The user's Guide to the General Health Questionnaire. Windsor: NFER-Nelson (1988).

[37] Zung W. A rating instrument for anxiety disorders. Psychosomatics (1971) 12:371–9. 10.1016/S0033-3182(71)71479-0 5172928

[38] Hamilton M. A rating scale for depression. J Neurol Neurosurg Psychiatry (1960) 23:56–62. 10.1136/jnnp.23.1.56 14399272 PMC495331

[39] Bernstein EM Putnam FW. Development, reliability, and validity of a dissociation scale. J Nerv Ment Dis. (1986) 174:727–35. 10.1097/00005053-198612000-00004 3783140

[40] Oldfield RC. The assessment and analysis of handedness: the Edinburgh Inventory. Neuropsychologia (1971) 9:97–113. 10.1016/0028-3932(71)90067-4 5146491

[41] Cox BJ Swinson RP. Instrument to assess depersonalization-derealization in panic disorder. Depr. Anxiety (2002) 15:172–5. 10.1002/da.10051 12112722

[42] Roberts DC Marcelli V Gillen JS Carey JP Della Santina CC Zee DS. MRI magnetic field stimulates rotational sensors of the brain. Curr Biol. (2011) 21:1635–40. 10.1016/j.cub.2011.08.029 21945276 PMC3379966

[43] Curthoys IS. The Interpretation of Clinical Tests of Peripheral Vestibular Function. Laryngoscope (2012) 122:1342–52. 10.1002/lary.23258 22460150

[44] Pfeiffer C Lopez C Schmutz V Duenas JA Martuzzi R Blanke O. Multisensory origin of the subjective first-person perspective: visual, tactile, and vestibular mechanisms. PLoS ONE (2013) 8:e61751. 10.1371/journal.pone.0061751 23630611 PMC3632612

[45] Lopez C Lenggenhager B Blanke O. How vestibular stimulation interacts with illusory hand ownership. Conscious Cogn. (2010) 19:33–47. 10.1016/j.concog.2009.12.003 20047844

[46] Garcia-Larrea L. The posterior insular-opercular region and the search of a primary cortex for pain. Neurophysiol Clin. (2012) 42:299–313. 10.1016/j.neucli.2012.06.001 23040701

[47] Parkes CM. Factors determining the persistence of phantom pain in the amputee. J Psychosom Res. (1973) 17: 97–108. 10.1016/0022-3999(73)90010-X 4741689

[48] Houghton AD Nicholls G Houghton AL Saadah E McColl L. Phantom pain: natural history and association with rehabilitation. Ann R Coll Surg Engl. (1994) 76:22–5. 8117013 PMC2502176

[49] Ehde DM Czerniecki JM Smith DG Campbell KM Edwards WT Jensen MP. Chronic phantom sensations, phantom pain, residual limb pain, and other regional pain after lower limb amputation. Arch Phys Med Rehabil. (2000) 81:1039–44 10.1053/apmr.2000.7583 10943752

[50] Smith DG Ehde DM Legro MW Reiber GE del Aguila M Boone DA Phantom limb, residual limb, and back pain after lower extremity amputations. Clin Orthop Relat Res. (1999) 361:29–38. 10.1097/00003086-199904000-00005 10212593

[51] Fisher K Hanspal RS. Phantom pain, anxiety, depression, and their relation in consecutive patients with amputated limbs: case reports. BMJ (1998) 316:903. 10.1136/bmj.316.7135.903 9552839 PMC28494

[52] Ephraim PL Wegener ST MacKenzie EJ Dillingham TR Pezzin LE. Phantom pain, residual limb pain, and back pain in amputees: results of a national survey. Arch Phys Med Rehab. (2005) 86:1910–9. 10.1016/j.apmr.2005.03.031 16213230

[53] Anderson RJ Freedland KE Clouse RE Lustman PJ. The prevalence of comorbid depression in adults with diabetes. Diabetes Care (2001) 24:1069–78. 10.2337/diacare.24.6.1069 11375373

